# Risk assessment and mitigation of hepatitis C virus RNA carryover contamination in a reflex testing algorithm

**DOI:** 10.1128/jcm.00256-26

**Published:** 2026-06-25

**Authors:** Victoria Stepanyants, Emily Hallock, Mary Kathryn Doud, Erika Harrington, Anthony Leung, Kamran Kadkhoda, Hannah Wang

**Affiliations:** 1 Cleveland Clinic Lerner College of Medicine at Case Western Reserve University 2546, Cleveland, Ohio, USA; 2 Department of Pathology and Laboratory Medicine, Cleveland Clinic Foundation 583189, Cleveland, Ohio, USA; 3 Department of Pharmacy, Cleveland Clinic Foundation 583189, Cleveland, Ohio, USA; 4 Department of Infectious Diseases, Cleveland Clinic Foundation 275069, Akron, Ohio, USA; St Jude Children's Research Hospital, Memphis, Tennessee, USA

**Keywords:** diagnostic techniques, health services administration, clinical laboratory information systems, laboratories, hepatitis viruses, hepacivirus

## Abstract

Laboratory-based diagnosis of active hepatitis C virus (HCV) infection currently requires a positive antibody test and a confirmatory RNA test. Many institutions in the United States continue to require two separate sample collections, partially due to concerns for carryover contamination. Reflexive RNA testing of antibody-positive samples reduces loss to follow-up, improves linkage to care, and is a recommended operational strategy by the Centers for Disease Control and Prevention. This study describes false positivity rates of reflexive RNA testing in a reference laboratory before and after introduction of a risk mitigation strategy that requires medical director review for reflexed HCV viral loads < 1,000 IU/mL (defined as low-positive). Initial HCV antibody testing was performed at one of five chemistry laboratories; reflex HCV RNA testing was performed using the cobas HCV assay on cobas 8800 (Roche) at a central reference laboratory. A total of 4,079 consecutive positive RNA results were included pre-intervention, and 4,433 were included post-intervention. All low-positive results (*N* = 157) were retrospectively adjudicated as true-positive (*N* = 66), false-positive (*N* = 38), or unknown (*N* = 53), based on clinical history and redraw results. The pre-intervention false-positive rate was 0.44%, which was significantly reduced to 0.14% post-intervention, *P* = 0.008. Median viral load for those clinically adjudicated as true-positive was significantly higher at 97 IU/mL (IQR: 24, 330 IU/mL) compared to 17 IU/mL (IQR: 14, 58 IU/mL) classified as false-positive or 16 IU/mL (IQR: 14, 62 IU/mL) classified as unknown. This study supports wider implementation of reflexive RNA testing for antibody-positive samples and provides a blueprint for a risk mitigation strategy.

**IMPORTANCE:**

Most patients infected with hepatitis C virus are unable to spontaneously clear the virus and develop chronic infection. Chronic hepatitis C virus (HCV) infection can lead to life-threatening complications including liver cirrhosis, liver failure, and liver cancer. Therefore, accurate diagnosis of hepatitis C infection and linkage to treatment is paramount. To increase linkage to care, current Centers for Disease Control and Prevention (CDC) guidelines recommend single-visit sample collection to test for antibodies and, if antibody positive, viral RNA. However, almost half of the laboratories do not use reflexive testing strategies, in part due to concerns for carryover contamination. Our study describes a low risk of false-positive RNA results and suggests a risk mitigation intervention to further minimize false positives. Our work reaffirms CDC guidelines and provides real-world data to encourage other facilities to follow a reflex testing algorithm to increase linkage to care.

## INTRODUCTION

Laboratory-based diagnosis of active hepatitis C virus (HCV) infection currently requires an initial HCV antibody screening test followed by a confirmatory HCV RNA test. Current Centers for Disease Control and Prevention (CDC) recommendations on testing for HCV encourage completion of both antibody and RNA testing from specimen(s) collected at a single visit (1). This guidance aims to reduce the need for repeated visits and therefore maximize the percentage of patients with active HCV infection that can be linked to care. Single-visit sample collection strategies include collecting a single specimen to be used for both antibody and RNA testing or collecting two separate specimens from a single venipuncture.

While collecting two specimens would eliminate the risk of carryover contamination, collection and storage of two tubes are impractical in most settings given the relatively low seroprevalence of HCV (1.7% in the United States) (2). Not only would most blood collected for RNA testing never be used, this two-tube strategy increases logistic complexity for the laboratory by introducing additional steps of extra tube labeling, storage, retrieval, and disposal. Reflex RNA testing from the same tube of blood used for antibody testing has been shown to increase the proportion of patients with positive HCV antibody tests who receive confirmatory HCV RNA testing, decreasing the rates of omitted RNA testing by as much as 76% and significantly improving linkage to care (3, 4). However, many public health laboratories in the United States have yet to adopt a reflex testing strategy, with as many as half of laboratories surveyed by the Association of Public Health laboratories reporting a lack of reflex testing strategy (5).

A potential reason for the delay in adoption of reflexive testing is concern for false positives resulting from carryover contamination. Carryover contamination is a potential concern with single-sample reflex testing, especially when fixed needles are used for sample transfer in upstream serology testing, with samples with viral loads < 1,000 IU/mL being of highest concern (6). Despite this, contamination rates are reportedly low, with contamination reported in 3.29% of HCV-negative samples loaded onto the cobas 6800 directly following a positive sample (7).

In this study, we assess the risk of carryover contamination within a centralized reference laboratory that performs reflex HCV RNA testing for five regional laboratories across two states. We then present an intervention aimed at screening of low positive (<1,000 IU/mL) results, which minimizes the risk of a false-positive result while still releasing positive results for patients with high pretest probability, maximizing the number of patients linked to care.

## MATERIALS AND METHODS

### HCV screening algorithm

The HCV screening protocol at Cleveland Clinic follows the Centers for Disease Control (CDC)-recommended testing sequence of HCV antibody testing followed by HCV RNA testing if reactive or equivocal. The same serum or plasma sample tube used for initial HCV antibody testing is reflexed for HCV RNA testing without another blood draw, allowing for single-visit sample collection. The initial HCV antibody test may be performed at one of five laboratories within the enterprise. The reflex HCV RNA testing is performed at a single laboratory using the cobas HCV assay on the cobas 8800 (Roche Diagnostics), which has a lower limit of detection of 15 international units (IU)/mL. When reported, results contain a qualitative HCV RNA Detected/Not detected interpretation, alongside viral load if detected in IU/mL and log IU/mL, and information about the assay method and linear range.

### Risk assessment for carryover contamination

Ten samples were used to evaluate the analytical process at five different regional labs (A–E) where HCV antibody testing is conducted. They comprised five positive samples and five negative samples. The five positive samples were pooled residual patient sera, each with an HCV RNA load of 1,000,000 IU/mL or greater. The five negative samples were pooled residual patient sera, each with HCV RNA not detected. Each pool was divided into 1.5–2 mL aliquots for each outside lab. Labs A–E were instructed to run the specimens in an alternating sequence to assess the carryover of HCV RNA.

These samples were tested on analyzers such as the cobas e 801 (Roche), Atellica IM (Siemens), and Alinity i (Abbott). The cobas e 801 and Atellica IM use disposable tips, while the Alinity i uses a fixed needle for specimen transfer. Labs A–C were in Ohio; Labs D and E were in Florida. Afterward, specimens were returned to the central Molecular Microbiology laboratory in Ohio and tested for HCV RNA using the cobas HCV assay on the cobas 8800 (Roche) as per the manufacturer’s instructions (8).

### Risk mitigation intervention

In June 2023, a new workflow was implemented to mitigate the risk of false-positive HCV RNA results due to carryover contamination. Per this new workflow, medical laboratory scientists were instructed to hold all reflexed HCV RNA results with viral load <1,000 IU/mL for medical director review prior to resulting. The medical director reviewed the patient’s history and approved release of positive HCV RNA results only if the patient had a history of untreated or partially treated active HCV or signs/symptoms compatible with acute hepatitis. If no history was available to corroborate the HCV RNA result, the test was canceled with a request for repeat collection of a dedicated specimen for HCV RNA testing. Due to human error in recognizing these rare events, the workflow was iteratively improved during the post-intervention period through addition and subsequent improvement of a laboratory information system flag designed to stop auto-verification of reflexed positive HCV RNA results <1,000 IU/mL and alert the laboratory scientist to request medical director review prior to resulting. The post-intervention period was analyzed with an “intent-to-treat” principle.

### Data collection

Records of HCV antibody and reflex RNA test results from serum or plasma specimens collected between March 2022 and December 2024 were extracted from existing microbiology laboratory data. Cancellation status, redraw status, patient sex, and patient age at collection were included. Additional variables were collected from the Electronic Health Record about patients with initial HCV RNA viral load results <1,000 IU/mL (low-positive). These variables included lifetime history of intravenous drug use (IVDU), history of prior known HCV infection, other patient-reported risk factors, history of direct antiviral acting agent (DAA) treatment, redraw date, and redraw HCV RNA viral load. A repeated HCV RNA test was considered a redraw if the specimen was collected within 90 days of the original result. All medical records were reviewed >90 days following the original low-positive result.

### Retrospective clinical adjudication

All low-positive results were adjudicated as false-positive, true-positive, or unknown through a manual review of electronic health records based on the following predetermined definitions. A false-positive result was defined as a low-positive result followed within 90 days by an undetectable HCV RNA result or a negative HCV antibody test. A true-positive result was defined as a low-positive result meeting at least one of the following criteria: (i) followed within 90 days by a positive HCV RNA result of any viral load or a successful genotype test; (ii) from a patient with known untreated chronic HCV infection; or (iii) from a patient undergoing DAA treatment at collection. A result was classified as unknown if it was a low-positive that did not meet the criteria for either a false-positive or true-positive result.

### Statistical analysis

Significance testing for differences in frequencies of categorical variables was conducted using chi-squared or Fischer’s exact test, depending on the expected frequencies. Significance testing for differences in continuous variables was conducted using the Wilcoxon Rank Sum and Kruskal-Wallis tests. The significance level used for all testing was 0.05. Pairwise comparisons were performed with Bonferroni adjustment. Values resulted as HCV RNA detected <15 IU/mL were assigned as 14 IU/mL for the analysis. Values resulted as >100,000,000 IU/mL were assigned as 100,000,001 IU/mL. Statistical analysis and image generation were performed using R (version 4.4.3), RStudio (version 2024.12.1), and JMP Pro 18.

## RESULTS

### Risk assessment for carryover contamination

All five samples from Labs B (Cobas e 801), Lab C (Atellica IM), and Lab E (Cobas e 801) were negative for HCV RNA, as expected. Lab A (Alinity i) and Lab D (Cobas e 801) each returned 1/5 (20%) samples that tested falsely positive for HCV RNA (Table 1). These 2/25 (8%) samples falsely positive for HCV RNA had viral loads of 43.7 and 59.9 IU/mL, respectively.

**TABLE 1:** Results of HCV RNA carryover evaluation on residual serum pools in chemistry labs A–E^*a*^

Regional lab	Chemistry analyzer (manufacturer)	Specimen transfer type	Negative pools with HCV RNA detected (%)
Lab A	Alinity i (Abbott)	Fixed needle	1/5 (20%)
Lab B	cobas e 801 (Roche)	Disposable tips	0/5 (0%)
Lab C	Atellica IM (Siemens)	Disposable tips	0/5 (0%)
Lab D	cobas e 801 (Roche)	Disposable tips	1/5 (20%)
Lab E	cobas e 801 (Roche)	Disposable tips	0/5 (0%)
Total	–	–	2/25 (8%)

^*a*^ HCV, hepatitis C virus; –, not applicable.

### Effect of risk mitigation intervention

**Fig 1:**
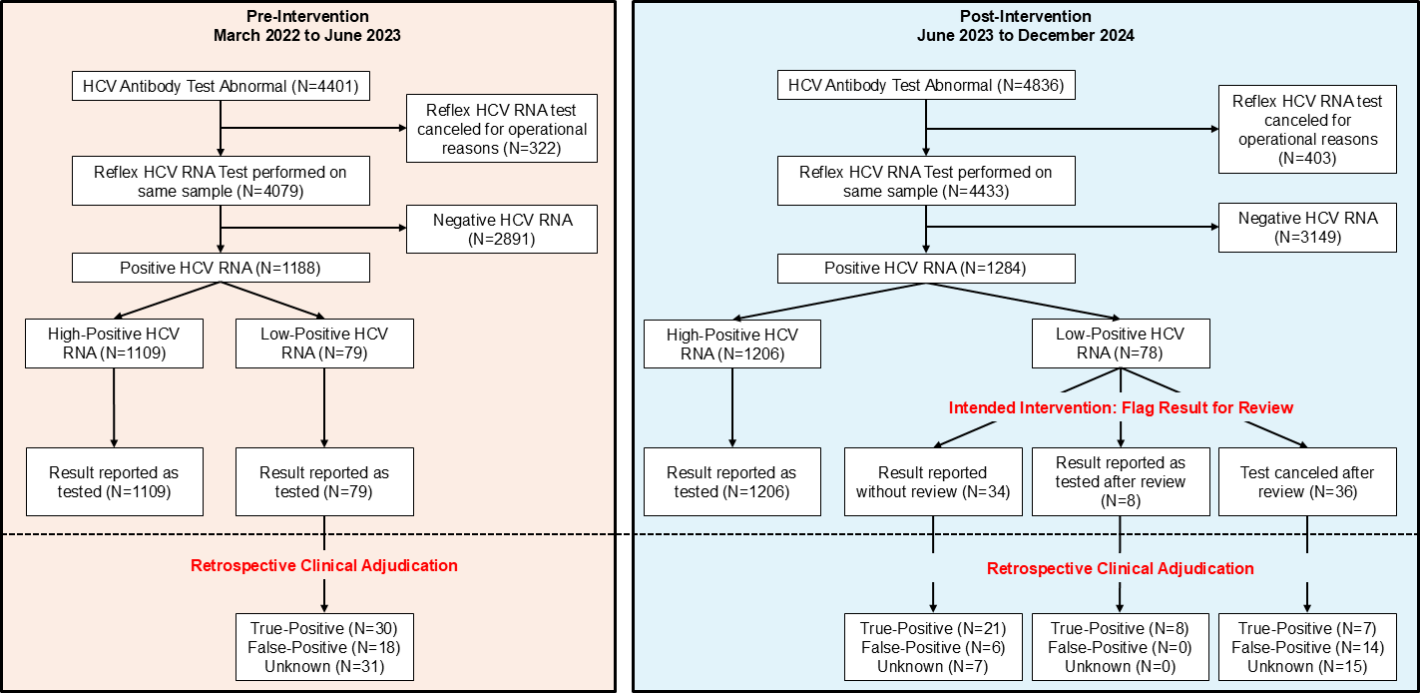
Flowchart depicting HCV RNA reflex testing workflow pre- (left) vs post- (right) risk mitigation intervention flagging low-positive HCV RNA results (<1,000 IU/mL) for review prior to resulting. Outcomes of retrospective clinical adjudication are provided below the dashed horizontal line.

**Fig 2:**
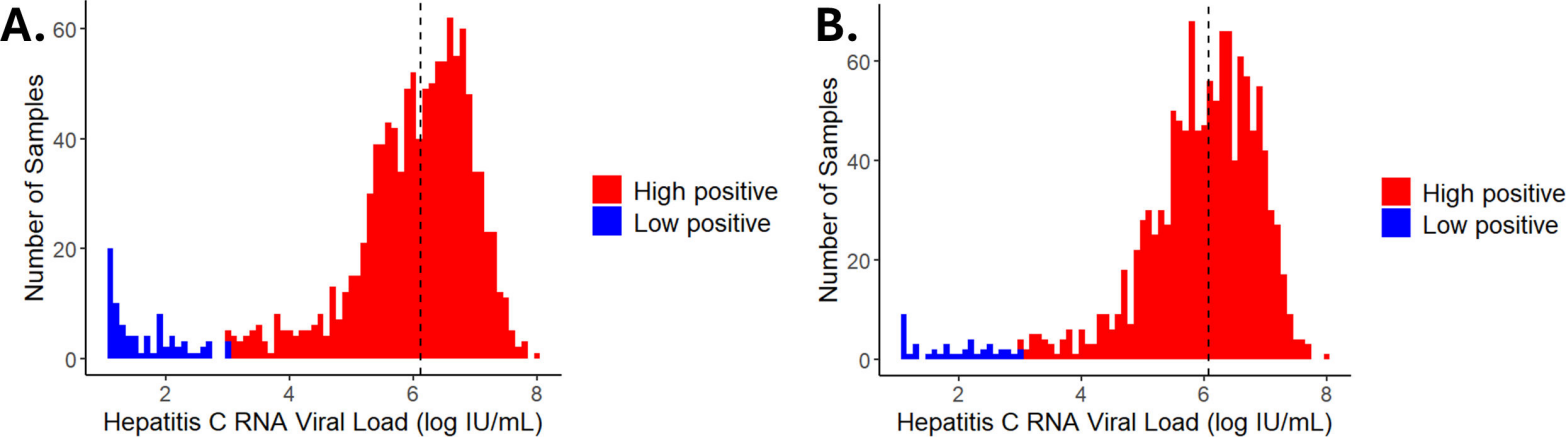
Histograms of reported HCV RNA viral loads pre-intervention (**A**) and post-intervention (**B**). The vertical dashed line represents the median viral load. Red bars highlight specimens with viral load ≥ 1,000 IU/mL (high positive), while blue bars highlight specimens with viral load < 1,000 IU/mL (low positive).

A total of 9,237 abnormal HCV antibody tests (8,555 positive/reactive, 682 equivocal) were studied across the pre-intervention and post-intervention time frames, with 91.8% of tests being successfully reflexed to HCV RNA testing (Fig. 1). Overall HCV RNA positivity rates and median viral load remained similar in the pre- vs post-intervention time periods (Table 2). Cancellations of reflex RNA testing were primarily due to the insufficient quantity of the remnant sample for testing. Among successfully performed and reported HCV RNA tests, the percentage of false-positive results decreased from 0.44% pre-intervention to 0.14% post-intervention (*P* = 0.008 and Table 2). The proportion of positive RNA tests with reported viral loads < 1,000 IU/mL was also significantly lower post-intervention (1.9% vs 0.96%, *P* < 0.001). However, the HCV RNA reflex test cancellation rate increased from 7.3% to 9.1% (*P* = 0.002). The viral loads of all results reported clinically pre-intervention and post-intervention are displayed in Fig. 2.

**TABLE 2:** Comparison of demographics, antibody, and reflex HCV RNA test results pre- and post-intervention among HCV antibody-abnormal samples^*a*^ (*N* = 9,237)

	All time (*N* = 9,237)	Pre-intervention (*N* = 4,401)	Post-intervention (*N* = 4,836)	*P* value
Female, *N* (%)	4,372 (47.3%)	2,067 (47.0%)	2,305 (47.7%)	0.47
Age at collection, median (IQR)	52 (37, 65)	51 (36, 65)	53 (38, 66)	<0.001
Antibody testing in Lab A, *N* (%)	7,909 (85.6%)	3,955 (89.9%)	3,954 (81.8%)	<0.001
Antibody testing in Lab B, *N* (%)	698 (7.6%)	300 (6.8%)	398 (8.2%)	0.01
Antibody testing in Lab C, *N* (%)	367 (4.0%)	136 (3.1%)	231 (4.8%)	<0.001
Antibody testing in Lab D, *N* (%)	70 (0.76%)	0 (0%)	70 (1.4%)	<0.001
Antibody testing in Lab E, *N* (%)	193 (2.1%)	10 (0.2%)	183 (3.8%)	<0.001
Reflexed HCV RNA tests resulted, *N* (%)	8,476 (91.8%)	4,079 (92.7%)	4,397 (90.9%)	0.002
Positive HCV RNA, *N* (%)	2,436 (28.7%)	1,188 (29.1%)	1,248 (28.4%)	0.45
HCV RNA viral load (log IU/mL), median (IQR)	6.09 (5.43, 6.65)	6.12 (5.42, 6.67)	6.07 (5.47, 6.63)	0.50
HCV RNA viral load < 1,000 IU/mL reported, *N* (%)	121 (1.43%)	79 (1.9%)	42 (0.96%)	<0.001
False-positive HCV RNA reported, *N* (%)	24 (0.28%)	18 (0.44%)	6 (0.14%)	0.008
Reflexed HCV RNA tests canceled, *N* (%)	761 (8.2%)	322 (7.3%)	439 (9.1%)	0.002

^*a*^ HCV, hepatitis C virus; IQR, interquartile range; IU, international units.

### Analysis of all low-positive HCV RNA results

There were 157 initially low-positive HCV RNA results identified between March 2022 and December 2024. Of those, 79 occurred before the implementation of the new workflow in June 2023, and the remaining 78 occurred after the implementation (Table 3). All pre-intervention low-positive results were reported clinically, with 18/79 (22.8%) of them determined to be false-positives after retrospective clinical adjudication. Two of these false-positive RNA tests were performed on samples initially reported as “equivocal” for HCV antibody.

**TABLE 3:** Comparison of initially low-positive (<1,000 IU/mL) HCV RNA test results pre- and post-intervention^*a*^ (*N* = 157)

	Pre-intervention reported (*N* = 79)	Post-intervention reported (*N* = 42)	Post-intervention canceled (*N* = 36)
HCV RNA load (IU/mL), median (IQR)	27.7 (14, 106)	92.4 (17.3, 277.5)	24.3 (14, 68.7)
HCV RNA load (log IU/mL), median (IQR)	1.50 (1.15, 2.06)	1.96 (1.24, 2.44)	1.43 (1.15, 1.84)
HCV RNA below quantification threshold < 15 IU/mL, *N* (%)	20 (25.3%)	9 (21.4%)	13 (36.1%)
Result flagged for review before reporting, *N* (%)	0 (0%)	8 (19.0%)	36 (100%)
True-positive, *N* (%)	30 (38.0%)	29 (69%)	7 (19.4%)
False-positive, *N* (%)	18 (22.8%)	6 (14.3%)^*b*^	14 (38.9%)
False-positive initially tested at Lab A, *N* (%)	18 (100%)	6 (100%)	14 (100%)
False-positive initially tested at Labs B/C/D/E, *N* (%)	0 (0%)	0 (0%)	0 (0%)
Unknown, *N* (%)	31 (39.2%)	7 (16.7%)	15 (41.7%)

^*a*^ HCV, hepatitis C virus; IU, international unit; IQR, interquartile range.

^*b*^ All six false-positive results reported in the post-intervention cohort were due to the low-positive result not being appropriately flagged for review.

In the post-intervention time period, only 44/78 (56.4%) low-positive results were flagged for clinical review as intended prior to resulting. The intervention failures were due to a mixture of human error and a fault in the laboratory information system flag build that was discovered retrospectively. All six post-intervention false-positives reported were done so erroneously without review due to intervention failure. In contrast, all eight positives reported after review were true-positives. Of the 36 low-positives that were canceled after medical director review, seven (19.4%) were deemed true-positives, 14 (38.9%) were deemed false-positives, and 15 (41.7%) were undetermined. One false-positive RNA test was performed on a sample initially reported as antibody “equivocal.” Of those with canceled tests, 15/36 (41.7%) did not present for redraw within 90 days.

All false-positive results across both time periods were initially tested at Lab A, which used a chemistry analyzer with fixed-needle transfer. Viral loads and proportion of results that were below the quantification threshold are summarized in Table 3. Patient characteristics are summarized in Table 4. The majority (75.8%) of low-positives adjudicated as true-positives had a history of prior HCV infection and had a known history of IV drug use (66.7%). All patients with results adjudicated as unknown did not present for redraw within 90 days of the initial low-positive result.

**TABLE 4:** Comparison of clinical characteristics of individuals with initially low-positive (<1,000 IU/mL) HCV RNA test results by retrospective clinical adjudication status^*a*^ (*N* = 157)

	True-positive (*N* = 66)	False-positive (*N* = 38)	Unknown (*N* = 53)
Demographics			
Female, *N* (%)	33 (50.0%)	20 (54.1%)	24 (45.3%)
Age, median (IQR)	43 (35.8, 60.3)	56 (34, 67.5)	41 (33, 55)
Criteria for clinical adjudication			
Negative HCV RNA/antibody test on redraw, *N* (%)^*b*^	9 (23.1%)	38 (100%)	0 (0%)
Positive HCV RNA or genotype on redraw, *N* (%)^*b*^	30 (45.5%)	0 (0%)	0 (0%)
No redraw within 90 days, *N* (%)	23 (34.8%)	0 (0%)	53 (100%)
Known untreated chronic HCV infection, *N* (%)	34 (51.5%)	0 (0%)	0 (0%)
Actively undergoing DAA treatment, *N* (%)	7 (10.6%)	0 (0%)	0 (0%)
Risk factors for HCV infection			
Symptoms within 90 days prior, *N* (%)	34 (51.5%)	7 (18.4%)	7 (13.2%)
History of IVDU, *N* (%)	44 (66.7%)	4 (10.5%)	25 (47.2%)
Other patient-reported exposure, *N* (%)^*c*^	10 (15.1%)	2 (5.3%)	2 (3.8%)
Prior HCV infection (any time), *N* (%)	50 (75.8%)	11 (28.9%)	17 (32.1%)
History of DAA (any time), *N* (%)	16 (24.2%)	7 (18.4%)	5 (9.4%)

^*a*^ IU, international unit; HCV, hepatitis C virus; IQR, interquartile range; DAA, direct-acting antiviral; IVDU, intravenous drug use.

^*b*^ Within 90 days of initial low-positive HCV RNA test.

^*c*^ Other known patient-reported risk factors included tattoos received in prison (1), tattoos received in places with questionable sanitary practices (4), sexual contact with a HCV-positive individual (2), blood transfusion before 1992 (1), needle stick in a healthcare worker (1), organ transplant from a HCV-positive donor (2), and contact with blood from a HCV-positive person (1).

### Optimal viral load threshold for risk mitigation

**Fig 3:**
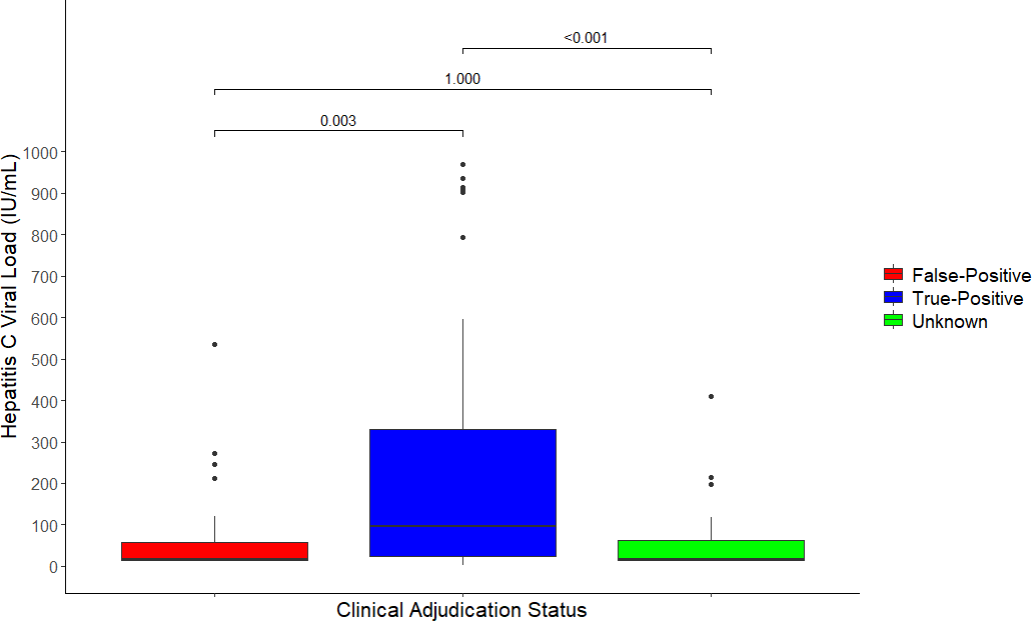
Box and whisker plots of HCV RNA viral loads by the clinical adjudication status (false-positive = red, left [*N* = 38], true-positive = blue, middle [*N* = 66], and unknown = green, right [*N* = 53]) for initially low-positive (<1,000 IU/mL) results. Median viral load for those clinically adjudicated as true-positives was significantly higher at 97 IU/mL (IQR: 24, 330 IU/mL) compared to 17 IU/mL (IQR: 14, 58 IU/mL) from those classified as false-positives or 16 IU/mL (IQR: 14, 62 IU/mL) classified as Unknown. *P* values are provided above the brackets for each pairwise comparison.

Viral loads were significantly different between true-positive, false-positive, and unknown low-positive results (Fig. 3). Viral load distribution of true-positive results had a wider IQR and was more positively skewed than false-positive and unknown results. The threshold initially chosen for the risk mitigation strategy was 1,000 IU/mL based on data from the risk assessment and literature (6, 7). The highest observed false-positive result in our study was 535 IU/mL, which supports the choice of the initial threshold at 1,000 IU/mL having near-100% sensitivity for detection of false-positives. The probability of a viral load result <1,000 IU/mL being a false-positive was 36.5% (38/104). At <100 IU/mL, probability of a false-positive result increased to 51.6% (33/64), but this reduced threshold would have resulted in erroneously reporting 13.2% (5/38) of false-positives. With a threshold of <25 IU/mL, probability of a false-positive result was 61.9% (26/42), and probability of erroneously reporting a false-positive result would be 31.6% (12/38). At <15 IU/mL, probability of a false-positive result increased further to 82.6% (19/23), with 50% (19/38) of false-positives missed.

## DISCUSSION

In this study, we evaluate the risk of HCV RNA carryover contamination associated with reflex testing from residual HCV antibody-abnormal sera and describe the effectiveness of a mitigation strategy aimed at decreasing the total number of false-positives reported. Our findings demonstrate that with appropriate risk assessment and mitigation, the benefits of reflex HCV RNA testing can outweigh the risks of carryover contamination. They provide a blueprint to other labs seeking to implement the CDC’s updated operational guidance encouraging single-visit sample collection for HCV antibody and RNA testing utilizing a single sample (1).

Our contrived risk assessment evaluation with alternating high-positive and -negative samples tested revealed that HCV RNA carryover contamination affected 2/25 (8%) samples tested, both with viral loads < 100 IU/mL, like prior reports. One of these false positives came from lab D on an instrument using disposable pipette tips, demonstrating that carryover risk may not be limited to instruments using fixed-needle transfer, despite a previous risk assessment reporting a non-existent risk of carryover contamination by HCV, HBV, and HIV RNA using serology samples processed using disposable tips prior to RNA quantification by cobas 6800 (9). Other possible sources of contamination include nonsterile pre-analytic manual processing and splashes or drips in the chemistry analyzer. The contrived evaluation was conducted in lab D only a few months after assay go-live; the assay being relatively new to this lab could also have been a factor in specimen contamination. Interestingly, in clinical testing, false positives were only seen from chemistry lab A. This could be due to the greater risk of contamination from fixed-needle transfer but could also be due to the higher specimen volume, other practices unique to this laboratory, or the fact that this laboratory is in closest proximity to the HCV RNA testing lab, which increases the likelihood that low-level HCV RNA remains stable after transport.

As expected, the false-positive rate in routine clinical testing was much lower than seen in the contrived risk assessment. Even before implementation of the risk mitigation workflow, the false-positive rate was low (0.44%). With the risk mitigation workflow, the false-positive rate was further reduced (0.14%). Notably, this decrease was significant despite the intervention only being successfully implemented 56.4% (44/78) of the time. Indeed, all false-positive results reported post-intervention had failed to be flagged for medical director review. The intervention failures were initially due to human error in recognizing these rare events. To assist, a laboratory information system (LIS) flag was built to flag these reflexed low-positive results and prevent their auto-verification. Subsequent failures were identified, and it was discovered that the LIS flag logic had been predicated on auto-verification and that it did not appropriately fire when auto-verification was turned off. Failure of automated flag-based interventions due to logic errors and human error has been noted in other studies of HCV screening (10, 11). The logic of the LIS flag described in this study has subsequently been corrected. With higher-fidelity implementation, the false-positive rate from carryover contamination with this risk mitigation strategy could be reduced even further.

In our sample of low-positive results, 15 out of 36 (44.7%) patients with canceled initial low-positive results did not present for redraw within 90 days of initial sample collection. Assuming a similar attrition rate among the larger population, an additional 3,740 patients were able to receive confirmatory HCV RNA test results through reflexive testing over 33 months, due to this reflex testing strategy. Previously reported rates of failure to present for redraw in two-sample HCV testing paradigms range from 33% to 89%, depending on the country and specific patient population (12–16). Two meta-analyses concluded that reflex HCV RNA testing significantly increases HCV RNA testing uptake (4, 17). This effect is especially pronounced in at-risk populations, such as patients seen in the emergency department (11). Notably, there is a growing body of literature suggesting that reflex RNA testing facilitates linkage to care (18, 19). A study that assessed the implementation of reflex testing in Andalusia, Spain, found a significant increase in the proportion of patients referred to care and a significant decrease in the time between HCV diagnosis and follow-up care (20). A similar study conducted in Barcelona, Spain, found that reflex HCV testing allowed them to connect 45/69 (65.2%) of eligible RNA-positive patients to a hepatology clinic (21). Similarly, a recent meta-analysis focused on the uptake of HCV RNA testing found that, although data were limited, reflex testing may increase both linkage to care and treatment initiation (4).

Our chosen initial low-positive threshold of 1,000 IU/mL was based on limited previous literature. Each laboratory conducting reflexive testing may benefit from reviewing the characteristics of their identified false-positive results to determine an effective cutoff. The logistical and financial burden of identifying and reviewing these low-positive results should be considered but are expected to be lower than the alternative of drawing a second tube of blood for every patient (1). The viral loads of the false-positives in our study were significantly lower than those from specimens clinically adjudicated to be true-positives. Importantly, an HCV RNA detected result <25 IU/mL (the manufacturer-stated threshold to define the absence of active HCV infection) had a 61.9% (26/42) probability of being a false-positive result. After reviewing these data and strengthening of the LIS flagging rules, our laboratory has now shifted to a lower threshold of 100 IU/mL from reflexed specimens to trigger test cancellation and redraw request without requiring medical director review.

Current American Association for the Study of Liver Diseases–Infectious Disease Society of America guidelines recommend a simplified HCV treatment regimen for treatment-naïve adults without cirrhosis, which includes a quantitative HCV RNA test any time prior to starting DAA (22, 23). Professional societies could consider recommending the incorporation of a viral load threshold for redraw and repeat HCV RNA testing to confirm active HCV infection to mitigate concerns for carryover contamination. Such a recommendation may help promote more widespread adoption of reflex testing.

A major strength of our study is the assessment of HCV RNA carryover contamination risk from five different chemistry laboratories across the country, providing increased generalizability, as well as a risk assessment and mitigation blueprint for other labs that may serve as regional or national reference centers. Another strength of this study is the availability of detailed clinical history to more accurately adjudicate HCV RNA results as true- vs false-positives. Study weaknesses include inclusion of only a single HCV RNA testing platform, limiting generalizability to other HCV nucleic acid amplification tests. Additionally, the study is limited by the clinical criteria applied for adjudication of results as false- or true-positives. Individuals with low-level active HCV infection could have spontaneously cleared their HCV infection between tests and thus be wrongly classified as a false-positive. Alternatively, individuals who may have had an initial false-positive HCV RNA result but subsequently were newly infected between tests could be wrongly classified as a true-positive. Furthermore, this study did not exclude patients with previously positive HCV antibody results, which made adjudication not only more challenging but also more representative of the real-world implementation of reflex testing and the proposed mitigation strategy.

The results of the present study support the implementation of reflex HCV RNA testing by demonstrating an acceptably low false-positive rate in routine clinical testing and describing an effective risk mitigation strategy to address the concerns of carryover contamination with the potential to reduce the false-positive rate to 0%. They provide a blueprint for other clinical laboratories seeking to implement reflex HCV RNA testing while maintaining high quality and fulfilling regulatory requirements.

## Data Availability

De-identified data are available from the corresponding author, H.W., upon reasonable request.

## References

[1] Cartwright EJ, Patel P, Kamili S, Wester C. 2023. Updated operational guidance for implementing CDC’s recommendations on testing for hepatitis c virus infection. MMWR Morb Mortal Wkly Rep 72:766–768. doi:10.15585/mmwr.mm7228a2 37440452 PMC10360608

[2] Hofmeister MG, Rosenthal EM, Barker LK, Rosenberg ES, Barranco MA, Hall EW, Edlin BR, Mermin J, Ward JW, Ryerson AB. 2019. Estimating prevalence of hepatitis c virus infection in the United States, 2013-2016. Hepatology 69:1020–1031. doi:10.1002/hep.30297 30398671 PMC6719781

[3] López-Martínez R, Arias-García A, Rodríguez-Algarra F, Castellote-Bellés L, Rando-Segura A, Tarraso G, Vargas-Accarino E, Montserrat-Lloan I, Blanco-Grau A, Caballero-Garralda A, Ferrer-Costa R, Pumarola-Sunye T, Buti-Ferret M, Esteban-Mur R, Quer J, Casis-Saez E, Rodríguez-Frías F. 2019. Significant improvement in diagnosis of hepatitis c virus infection by a one-step strategy in a central laboratory: an optimal tool for hepatitis c elimination? J Clin Microbiol 58:e01815-19. doi:10.1128/JCM.01815-19 31694971 PMC6935937

[4] Tao Y, Tang W, Fajardo E, Cheng M, He S, Bissram JS, Hiebert L, Ward JW, Chou R, Rodríguez-Frías F, Easterbrook P, Tucker JD. 2023. Reflex hepatitis c virus viral load testing following an initial positive hepatitis c virus antibody test: a global systematic review and meta-analysis. Clinical Infectious Diseases 77:1137–1156. doi:10.1093/cid/ciad126 37648655

[5] Johnson LN, Gaynor AM, Wroblewski K, Buss SN. 2024. Maximizing reflexive hepatitis c virus (HCV) RNA testing of HCV antibody-reactive samples within United States public health laboratories. J Infect Dis 229:S357–S361. doi:10.1093/infdis/jiad191 37739790 PMC11078303

[6] Rondahl E, Gruber M, Joelsson S, Sundqvist M, Åkerlind B, Cardell K, Lindh M, Serrander L. 2014. The risk of HCV RNA contamination in serology screening instruments with a fixed needle for sample transfer. J Clin Virol 60:172–173. doi:10.1016/j.jcv.2014.03.011 24735614

[7] Thompson LA, Fenton J, Charlton CL. 2022. HCV reflex testing: a single-sample, low-contamination method that improves the diagnostic efficiency of HCV testing among patients in Alberta, Canada. J Assoc Med Microbiol Infect Dis Can 7:97–107. doi:10.3138/jammi-2021-0028 36337352 PMC9608109

[8] FDA. 2015. Assay, hybridization and/or nucleic acid amplification for detection of hepatitis c rna, hepatitis c virus. Available from: https://www.accessdata.fda.gov/scripts/cdrh/cfdocs/cfpma/pma.cfm?id=P150015

[9] Rodriguez PL, McCune S, Sakai L, Engstrom-Melnyk J, Marins E. 2020. Evaluation of contamination risk by the cobas e 602 serology module before viral load testing on the cobas 6800 system. Sex Transm Dis 47:S32–S34. doi:10.1097/OLQ.0000000000001125 31895305

[10] Schechter-Perkins EM, Miller NS, Hall J, Hartman JJ, Dorfman DH, Andry C, Linas BP. 2018. Implementation and preliminary results of an emergency department nontargeted, opt-out hepatitis c virus screening program. Acad Emerg Med 25:1216–1226. doi:10.1111/acem.13484 29851238 PMC6957127

[11] Manteuffel JJ, Lee MS, Bussa RM, Sabagha NL, Chaudhry K, Ross JE, Cook MR, Rammal J-AK, Sridasyam K, Klausner HA, Samuel LP, Miller JB. 2022. Hepatitis c virus reflex testing protocol in an emergency department. West J Emerg Med 23:108–114. doi:10.5811/westjem.2021.10.52468 35302440 PMC8967471

[12] Yartel AK, Morgan RL, Rein DB, Ann Brown K, Kil NB, Massoud OI, Fallon MB, Smith BD. 2015. HIV infection status as a predictor of hepatitis c virus RNA testing in primary care. Am J Prev Med 49:423–427. doi:10.1016/j.amepre.2015.03.003 25896194 PMC4556132

[13] McGibbon E, Bornschlegel K, Balter S. 2013. Half a diagnosis: gap in confirming infection among hepatitis c antibody-positive patients. Am J Med 126:718–722. doi:10.1016/j.amjmed.2013.01.031 23786667

[14] Rupasinghe D, Choi JY, Kumarasamy N, Pujari S, Khol V, Somia IKA, Lee MP, Pham TN, Kiertiburanakul S, Do CD, Avihingsanon A, Ross J, Jiamsakul A, on behalf the international epidemiology databases to evaluate AIDS (IeDEA) Asia‐Pacific. 2024. Post-diagnosis HCV RNA testing rates prior to HCV treatment among people living with HIV with HCV antibody positivity in the Asia-Pacific Region. J Viral Hepat 31:686–699. doi:10.1111/jvh.13993 39115260 PMC11496006

[15] Morales-Arraez D, Alonso-Larruga A, Diaz-Flores F, García Dopico JA, de Vera A, Quintero E, Hernández-Guerra M. 2019. Predictive factors for not undergoing RNA testing in patients found to have hepatitis c serology and impact of an automatic alert. J Viral Hepat 26:1117–1123. doi:10.1111/jvh.13122 31077515

[16] Rongey CA, Kanwal F, Hoang T, Gifford AL, Asch SM. 2009. Viral RNA testing in hepatitis c antibody-positive veterans. Am J Prev Med 36:235–238. doi:10.1016/j.amepre.2008.10.013 19162434

[17] Mathews R, Shen C, Traeger MW, O’Brien HM, Roder C, Hellard ME, Doyle JS. 2025. Enhancing hepatitis c virus testing, linkage to care, and treatment commencement in hospitals: a systematic review and meta-analysis. Open Forum Infect Dis 12:ofaf056. doi:10.1093/ofid/ofaf056 39935959 PMC11811904

[18] Coyle C, Viner K, Hughes E, Kwakwa H, Zibbell JE, Vellozzi C, et al.. 2012. Identification and linkage to care of HCV-infected persons in five health centers. Vol. 64. 25950252, Philadelphia, Pennsylvania. PMC4584550 25950252

[19] Rhea S, Seña AC, Hilton A, Hurt CB, Wohl D, Fleischauer A. 2018. Integrated hepatitis c testing and linkage to care at a local health department sexually transmitted disease clinic: determining essential resources and evaluating outcomes. Sex Transm Dis 45:229–232. doi:10.1097/OLQ.0000000000000748 29465696

[20] Casas M de la P, García F, Freyre-Carrillo C, Montiel N, de la Iglesia A, Viciana I, Domínguez A, Guillot V, Muñoz A, Cantudo P, et al.. 2020. Towards the elimination of hepatitis C: implementation of reflex testing in Andalusia. Rev Esp Enferm Dig 112:515–519. doi:10.17235/reed.2020.6370/2019 32188257

[21] Llaneras J, Ruiz-Cobo JC, Rando-Segura A, Barreira-Díaz A, Domínguez-Hernández R, Rodríguez-Frías F, Campins M, Colom J, Casado MA, Blanco-Grau A, Bañares J, Monforte A, Falcó-Roget A, Ruíz L, Meza B, Pumarola T, Riveiro-Barciela M, Esteban R, Buti M. 2024. Integrating viral hepatitis management into the emergency department: a further step towards viral hepatitis elimination. JHEP Reports 6:100932. doi:10.1016/j.jhepr.2023.100932 38074506 PMC10698271

[22] Bhattacharya D, Aronsohn A, Price J, Lo Re V III, Heald J, Demisashi G, Durzy E, Davis-Owino A, Tynes S, the American association for the study of liver diseases–infectious diseases society of America HCV guidance panel. 2023. Hepatitis c guidance 2023 update: American association for the study of liver diseases– infectious diseases society of America recommendations for testing, managing, and treating hepatitis c virus infection. Clin Infect Dis. doi:10.1093/cid/ciad319

[23] Ghany MG, Morgan TR, Guidance P. 2020. Hepatitis c guidance 2019 update: American association for the study of liver diseases-infectious diseases society of america recommendations for testing, managing, and treating hepatitis c virus infection. Hepatology 71:686–721. doi:10.1002/hep.31060 31816111 PMC9710295

